# The melanocyte inducing factor MITF is stably expressed in cell lines from human clear cell sarcoma

**DOI:** 10.1038/sj.bjc.6601212

**Published:** 2003-09-09

**Authors:** K K C Li, J Goodall, C R Goding, S-K Liao, C-H Wang, Y-C Lin, H Hiraga, T Nojima, K Nagashima, K-L Schaefer, K A W Lee

**Affiliations:** 1 Department of Biology, HK University of Science & Technology, Kowloon, HK, China; 2 Marie Curie Research Institute, The Chart, Oxted, Surrey RH8 OTL, UK; 3 Graduate Institute of Clinical Medicine, Chang Gung University, Kweishan, Taoyuan 333, Taiwan; 4 Division of Hematology/Oncology, Department of Internal Medicine, Chang Gung Memorial Hospital, Kweishan, Taoyuan 333, Taiwan; 5 Division of Orthopaedics, Department of Clinical Research, National Sapporo Hospital, Sapporo 003-0804, Japan; 6 Department of Clinical Pathology, Kanazawa Medical University, Ishikawa 920-0293, Japan; 7 Department of Pathology, Hokkaido University School of Medicine, N-15, W-7, Kita-ku, Sapporo, Japan; 8 Institute of Pathology, Heinrich-Heine-University, Dusseldorf, Germany

**Keywords:** EWS/ATF1, MITF, melanocytes, clear cell sarcoma

## Abstract

Clear cell sarcoma (CCS) is associated with the EWS/ATF1 oncogene that is created by chromosomal fusion of the Ewings Sarcoma oncogene (EWS) and the cellular transcription factor ATF1. The melanocytic character of CCS suggests that the microphthalmia-associated transcription factor (Mitf), a major inducer of melanocytic differentiation, may be miss-expressed in CCS. Accordingly, we show that the mRNA and protein of the melanocyte-specific isoform of Mitf (Mitf-M) are present in several cultured CCS cell lines (Su-ccs-1, DTC1, Kao, MST-1, MST-2 and MST-3). The above cell lines thus provide a valuable experimental resource for examining the role of Mitf-M in both CCS and melanocyte differentiation. Melanocyte-specific expression of Mitf-M is achieved via an ATF-dependent melanocyte-specific cAMP-response element in the Mitf-M promoter, and expression of Mitf-M in CCS cells suggests that EWS/ATF1 (a potent and promiscuous activator of cAMP-inducible promoters) may activate the Mitf-M promoter. Surprisingly, however, the Mitf-M promoter is not activated by EWS/ATF1 in transient assays employing CCS cells, melanocytes or nonmelanocytic cells. Thus, our results indicate that Mitf-M promoter activation may require an appropriate chromosomal context in CCS cells or alternatively that the Mitf-M promoter is not directly activated by EWS/ATF1.

Human clear cell sarcoma (CCS) is a rare aggressive tumour, which is typically associated with tendons and aponeuroses and is thought to be of neuroectodermal origin (Chung and Enzinger 1983; Epstein *et al*, 1984). Clear cell sarcoma has also been called malignant melanoma of soft parts (MMSP) due to the occurrence of melanocytic markers and in some cases pigmentation (Epstein *et al*, 1984). Molecular studies of CCS have been limited due to the rarity of this malignancy and diagnostic problems prior to identification of the EWS/ATF1 oncogene as the definitive molecular signature of CCS.

EWS/ATF1 is created by aberrant t(12;22)(q13;q12) chromosomal fusion (Zucman *et al*, 1993) of the Ewings Sarcoma oncogene (EWS) to the C-terminal region of activating transcription factor 1 (ATF1) . Activating transcription factor 1 is a bZIP protein that mediates cAMP-inducible transcription (Ribeiro *et al*, 1994), while in contrast EWS/ATF1 is a potent constitutive activator of cAMP-inducible promoters (Brown *et al*, 1995; Fujimura *et al*, 1996; Pan *et al*, 1998; Feng and Lee, 2001); thus, suggesting that EWS/ATF1 may deregulate transcription of cAMP-inducible promoters in CCS cells. The physiological target promoters for EWS/ATF1 and their potential role in determining CCS biology and malignant transformation are beginning to be identified (Schaefer *et al*, 2002; Jishage *et al*, 2003). In addition to having a role in CCS genesis, EWS/ATF1 appears to be involved in CCS maintenance (Bosilevac *et al*, 1999) and may therefore be a therapeutic target.

The pigmentation that is sometimes associated with CCS suggests that ectopic expression of the melanocyte-inducing factor Mitf (microphthalmia-associated transcription factor; reviewed by Goding 2000) might be a key feature of CCS. Microphthalmia-associated transcription factor exists in multiple isoforms (Mitf-A, Mitf-B, Mitf-C, Mitf-D, Mitf-H and Mitf-M) that are all members of the basic helix–loop–helix/leucine zipper (bHLH/LZ) family of transcription factors. The Mitf isoforms are highly related (Figure 3A

**Figure 3:**
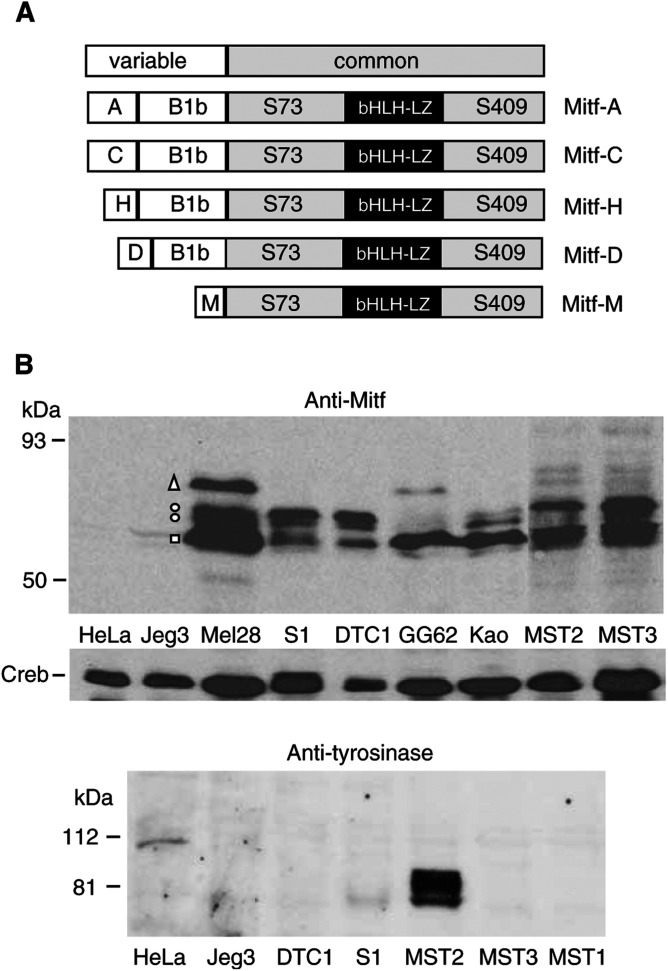
Expression of Mitf-M and tyrosinase proteins in CCS cells. (**A**) Structure of different isoforms of Mitf. White boxes represent the n-terminal exons (including the translation initiation site) unique to each isoform and the grey box is common to all isoforms. The black box shows the position of the bHLH-LZ DNA binding and dimerisation domain of Mitf. S73 and S409 are serine phosphoacceptor sites for MAPK and Rsk-1, respectively. (**B**) Detection of Mitf and tyrosinase by Western blotting. For Mitf, whole-cell extracts from Mel28 cells (positive control), the CCS cells indicated or Jeg3 and HeLa cells (negative control) were analysed by Western Blotting using anti-Mitf antibody (NeoMarkers AB-1 (C5)). Different Mitf isoforms are indicated by an open box, open circles and an open triangle adjacent to the Mel28 melanoma cell sample. Molecular weight standards (kDa, Biorad prestained low molecular weight range) are indicated to the left. The same samples were also probed with anti-CREB antibody (Cat #9192, New England Biolabs) as a positive control. For tyrosinase, whole-cell extracts were probed with antityrosinase antibody (C19, Santa Cruz) and molecular weight standards (kDa, Biorad prestained low molecular weight range) are indicated to the left.

), but contain unique n-terminal exons expressed from distinct promoters (Udono *et al*, 2000, Figure 2A

**Figure 2:**
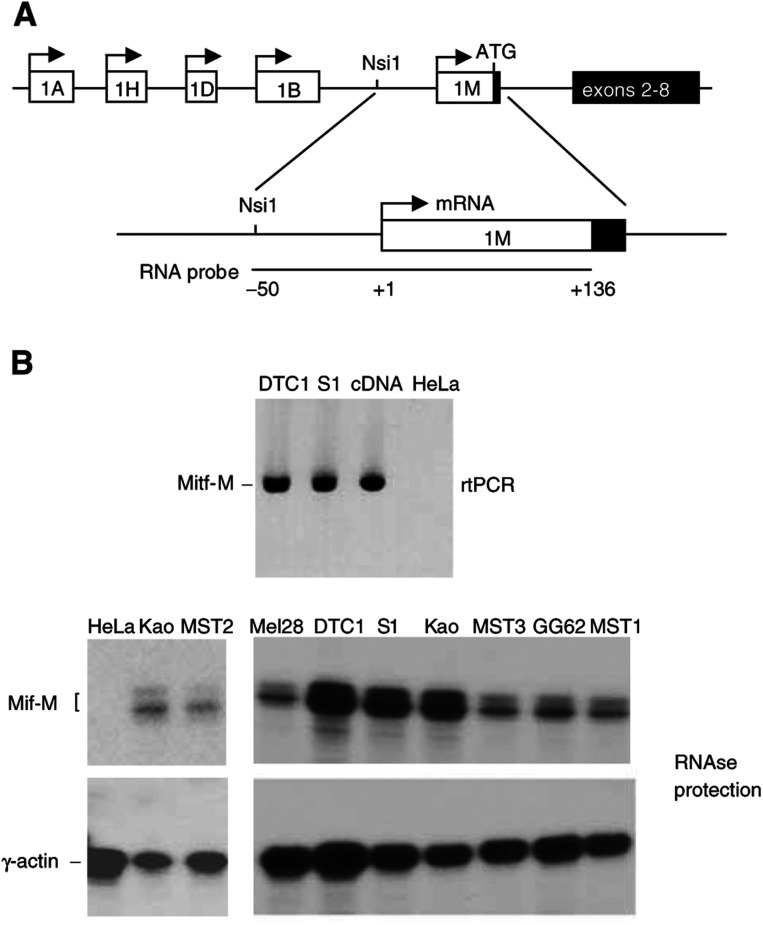
Analysis of Mitf-M RNA expression. (**A**) Structure of the human Mitf (hMitf) gene. The hMitf gene has alternative first exons (1A, 1 H, 1D, 1B and 1 M, shown in open boxes and 1C, not shown or mapped) each containing a translation initiation codon, which arise from the use of five different promoters. Fusion of alternative first exons to common downstream exons 2–8 (black box) gives rise to distinct isoforms of Mitf (Mitf-A, Mitf-H, Mitf-D, Mitf-C, Mitf-B and Mitf-M) that differ only in their extreme n-terminal protein sequences. The Mitf-M promoter is melanocyte specific. (**B**) Detection of Mitf-M transcripts in CCS cells. Total RNA was extracted from the CCS cell lines indicated (S1(Su-ccs-1)) and negative control (HeLa)) or positive control (Mel28) cells. RNA samples were analysed by RT–PCR (upper panel) or by RNAse protection assay (lower panel). microphthalmia-associated transcription factor-M isoform-specific primers used for RT–PCR analysis are described in Materials and Methods. ^32^P-labelled antisense probes for Mitf-M (shown in part A) and *γ*-actin as invariant control, were used for RNAse protection assays. Bands indicated to the left of the autoradiogram are the ^32^P-labelled protected fragments corresponding to correctly initiated Mitf-M and *γ*-actin mRNAs.

) that yield different expression profiles. The Mitf-M isoform is melanocyte specific and can induce melanocytic differentiation (Tachibana *et al*, 1996), indicating that Mitf-M is a critical melanogenic factor. Microphthalmia-associated transcription factor acts in part via direct transcriptional activation of genes (tyrosinase, TYRP1 and TYRP2) required for pigment synthesis (Bentley *et al*, 1994; Hemesath *et al*, 1994; Yavuzer *et al*, 1995; Yasumoto *et al*, 1997) and also has a role in melanocyte survival and differentiation (Lerner *et al*, 1986; Opdecamp *et al*, 1997; Hemesath *et al*, 1998). With respect to CCS, it is significant that transcription of the Mitf-M isoform is controlled by a melanocyte-specific promoter (Fuse *et al*, 1996) that includes a cAMP-response element (CRE) comprising a single ATF1/CREB binding site (Bertolotto *et al*, 1998). Thus, the Mitf-M promoter is a potential target for EWS/ATF1.

Limited attempts have been made to examine the Mitf-M expression in CCS. One study based on immunostaining of tumour specimens (Granter *et al*, 2001) concluded that Mitf is a marker for CCS, but did not confirm expression of the Mitf-M isoform due to detection method employed and use of the D5 Mitf-antibody (Hemesath *et al*, 1998) that recognises all Mitf isoforms. A second study detected Mitf-M RNA in nonviable CCS tumour material and did not examine Mitf-M protein expression (Antonescu *et al*, 2002). Here we show that Mitf-M mRNA and protein are expressed at significant levels in a wide range of CCS cell lines, demonstrating that Mitf-M is a stable marker for CCS in cell culture. In contrast, tyrosinase is not generally expressed in the CCS cells studied, indicating that elements of the pigmentation process downstream of Mitf are not stable in culture. Although the endogenous Mitf-M promoter is activated in CCS cells, the Mitf-M promoter is not active when transiently introduced into CCS cells or upon cotransfection with EWS/ATF1 in melanocytes or other cell types. Our results therefore indicate that Mitf-M promoter activation by EWS/ATF1 requires an appropriate chromosomal context in CCS cells or alternatively, that the Mitf-M promoter is not directly activated by EWS/ATF1.

## MATERIALS AND METHODS

### Cell culture and cell lines

**Figure 1:**
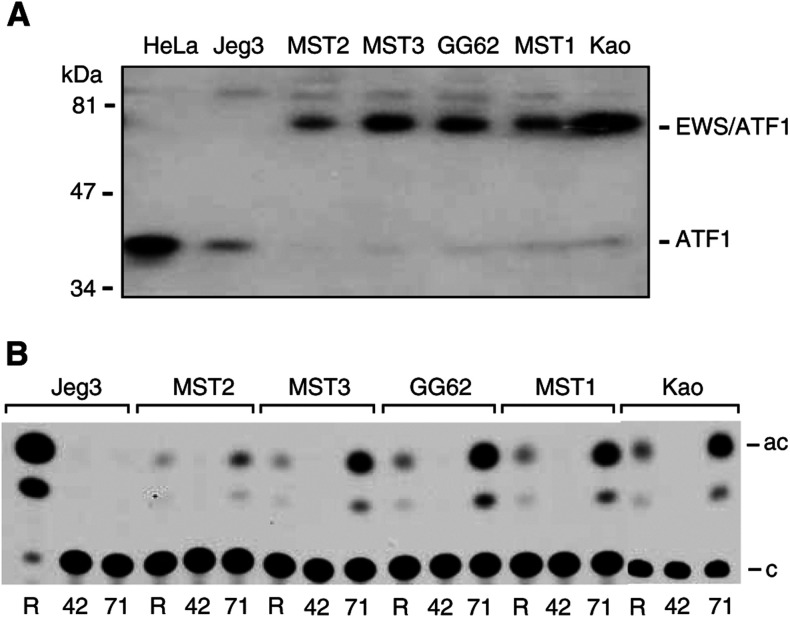
(**A**) Expression of EWS/ATF1 in CCS cell lines. Proteins present in nuclear extracts from multiple CCS cell lines (MST-1, MST-2, MST-3, GG62 and Kao) or HeLa and Jeg3 negative control cells were purified by ATF1-sequence-specific DNA-affinity chromatography and subjected to Western blot analysis using anti-ATF1 antibody. The ATF1 and EWS/ATF1 are indicated to the right and molecular weight standards (Biorad prestained, low molecular weight range) to the left. (**B**) ATF-site-dependent promoter activity in CCS cell lines. Transient transfection of CCS cell lines (indicated above) or Jeg3 control cells lacking EWS/ATF was performed using pRSVcat (R), pΔ(−71)SomCat containing a single ATF binding site (71) or pΔ(−42)SomCat lacking the ATF binding site (42). Transfection conditions were as described in the Materials and Methods and CAT assays were performed at 40 h post-transfection. A representative autoradiogram of a CAT assay is shown.

All cell lines were maintained as monolayers in Dulbecco's modification of Eagle's medium (DMEM) containing 10% FCS. Su-ccs-1 (Epstein *et al*, 1984), DTC1 (Brown *et al*, 1995), Kao (Hiraga *et al*, 1997), MST-1 (Liao *et al*, 1996) and GG-62 (Schaefer *et al*, 2002) cells have been described previously. All of the above cells (including MST-1, confirmed by detection of the EWS/ATF1 fusion transcript by rtPCR (Stella Chan and KL, unpublished results), MST-2 and MST-3 (S-K Liao, unpublished results) contain the t(12;22) translocation characteristic of CCS and express EWS/ATF1 RNA. The expression of EWS/ATF1 protein in all of the above cell lines is shown here (Figure 1A

).

### Plasmids and constructions

pΔ(−71)SomCAT contains the somatostatin promoter to position −71, fused to the chloramphenicol acetyl transferase (CAT) coding sequences (Montminy *et al*, 1986). pΔ(−42)SomCAT was obtained by digestion of pΔ(−71)SomCAT with Aat2, blunting with T4 DNA polymerase and religation (Brown *et al*, 1995). pRSVCAT contains the RSV LTR linked to CAT as previously described (Gorman *et al*, 1982). pMITF-MCAT was obtained by insertion of CAT and MITF-M promoter sequence from the position −395 into pSuppCT (Yavuzer and Goding, 1994). pMCRE contains two ATF sites from the MITF-M promoter upstream of the RSV TATA box and transcription site linked to CAT. pMCRE was obtained by insertion of an oligonucleotide containing tandem copies of the sequence (agcaTGACGTCAagccagg, the ATF site is underlined) between positions −151 and −133 of the MITF-M promoter into the *Sac*1 site of pΔERSVCAT (Tsukada *et al*, 1987). pMCREm corresponds to pMCRE containing mutated ATF sites (TGAtaTCA) that are unable to bind ATF1 (Jones and Lee, 1991). pΔ287C has been described previously (Pan *et al*, 1998) and expresses a protein, containing the n-terminal 287 amino acids of EWS fused to ATF1, which is virtually identical to intact EWS/ATF1. pMMRP was obtained by insertion of an *Nsi*1/*Hind*3 fragment (from position −50 to +136 of the MITF-M promoter) into *Pst1/Hind3*-digested pGem3.

### Transfections and reporter assays

For CAT reporters, transfections were carried out by calcium phosphate coprecipitation and CAT assays were performed at 40 h post-transfection as previously described (Gorman *et al*, 1982). For promoter analysis, precipitates contained 5 *μ*g of reporter plasmid and 20 *μ*g of total DNA made up with pGem3 as carrier. For cAMP-induction, cells were cotransfected with reporter and pCMVC*α* expressing the catalytic subunit of PKA and stimulated by addition of 300 *μ*M cptcAMP to the culture medium at 16 h post-transfection. For quantitation of results, percent conversion of unacetylated to acetylated ^14^C-chloramphenicol under linear assay conditions was determined by excision of spots from the TLC plate and quantitation of radioactivity using a liquid scintillation counter. For luciferase reporters, cells were transfected using Fugene reagent (Boehringer-Mannheim) according to the manufacturer's instructions and firefly luciferase activity was determined at 48 h post-transfection.

### RNA analysis

For the RT–PCR, total RNA was reverse transcribed with AMV reverse transcriptase (Boehringer, Mannheim, Germany) followed by first-strand cDNA synthesis (Amersham cDNA synthesis kit) and PCR as described previously (Carreira *et al*, 1998). Reverse transcription–PCR analysis of Mitf-M was performed using MITF-M forward (5′ATGCTGGAAATGCTAGAATATAATC3′) and reverse (5′CAATCAGGTTGTGATTGTCC3′) primers. For RNAse protection assays, preparation of total cellular RNA and detection of specific transcripts by RNAse protection analysis was performed as previously described. (Zinn *et al*, 1983). For detection of Mitf-M mRNA, pMMRP was linearised with *Xba*1 and transcribed by SP6 RNA polymerase to produce a high specific activity ^32^P-labelled antisense probe (Figure 2A).

### Antibodies and western blotting

For Western blotting, CREB antibody (New England Biolabs Beverly MA., Cat #9192) was used at a 1:1000 dilution, ATF-1 antibody (Hurst *et al*, 1991] at 1:200 dilution, Mitf antibody (NeoMarkers Ab-1(C5)) at 1:500 dilution and tyrosinase antibody (C19, Santa Cruz, CA) for CREB (Amersham NA934) peroxidase-conjugated sheep anti-mouse (Amersham, NXA 931) for Mitf and peroxidase-conjugated anti-goat (Sigma, product #A5420) for tyrosinase. ECL detection reagents (Amersham RPN2106) were used according to the manufacturer's instructions.

### Nuclear extracts and affinity purification

Nuclear extracts were prepared as previously described (Hurst *et al*, 1990). For DNA affinity purification, 200 *μ*l of nuclear extract (derived from ∼10^7^ cells) was incubated at room temperature with 20 *μ*l of affinity resin (by resuspending the resin several times over a period of 30 min) in the presence of 4 *μ*g ml^−1^ poly [dI/dC]). After binding, the affinity resin was washed with two changes of buffer (20 mM HEPES pH 7.4; 100 mM KCl) and SDS–PAGE sample buffer was then added directly to the washed resin.

## RESULTS

### Presence of EWS/ATF1 and ATF-site-dependent promoter activity in CCS cells

A consistent t(12;22)(q13;q12) chromosomal translocation that produces the EWS/ATF1 fusion gene distinguishes CCS from cutaneous melanoma (van Roggen *et al* 1998). Several CCS cell lines have been established in culture and of these, Su-ccs-1 and DTC1 have been shown to express the EWS/ATF1 fusion protein (Brown *et al*, 1995). Several other cell lines used in this study (Kao, GG62, MST-2, MST-1 (see Materials and Methods) and MST-3) contain the EWS/ATF1 fusion gene, but have yet to be characterised for EWS/ATF1 protein expression. We tested for the presence of EWS/ATF1 in the above cell lines as previously described (Brown *et al*, 1995) by using ATF1 DNA-affinity purification of proteins present in nuclear extracts and detection by Western blotting using anti-ATF1 (Figure 1A). ATF1 is expressed at low levels in the CCS cells tested, while EWS/ATF1 (as indicated by an ∼80 kDa polypeptide that binds to the ATF1 DNA affinity resin, reacts with a c-terminal ATF1 antibody and is absent in HeLa and Jeg3 cells) is detected at similar levels in all CCS cells tested.

We have previously shown that promoters that can be activated by EWS/ATF1 in a cotransfection assay are constitutively active when transiently introduced into CCS cells (Brown *et al*, 1995). For example, the somatostatin promoter (Δ(−71)SomCAT) is highly active (260% of RSVCAT) in DTC1 cells while exhibiting only background levels of activity (0.03% of RSVCAT) in Jeg3 cells. Deletion of the ATF1 binding site in the somatostatin promoter (Δ(−42)SomCAT) greatly reduces activity, strongly suggesting that endogenous EWS/ATF1 in CCS cells is responsible for high somatostatin promoter under the above conditions (Brown *et al*, 1995). To further characterise the additional CCS cells (Kao, GG62, MST1, MST-2 and MST-3) used here , we tested the ability of these to activate the Δ(−71)SomCAT reporter (Figure 1B). Similar to DTC1 and Su-ccs-1 cells, Δ(−71)SomCAT was highly active (MST-2, 285% of RSVCAT; MST-3, 800%; GG62, 860%; MST1, 583%; KAO, 320%), while Δ(−42)SomCAT exhibited minimal activity in all of the above cell lines. In summary, the presence of similar levels of EWS/ATF1 and the ability to support constitutive ATF-site-dependent promoter activity indicates that the CCS cells described should be useful for transcriptional studies of EWS/ATF1.

### Expression of Mitf-M RNA in CCS cells

The Mitf gene is controlled by multiple promoters (Udono *et al*, 2000, Figure 2A) that give rise to closely related but distinct isoforms (Mitf-A, Mitf-B, Mitf-C, Mitf-D, Mitf-H and Mitf-M, Figure 3A) that are distinguished by the presence of unique n-terminal exons. The Mitf-M expression is restricted to neural crest-derived melanocytes due to a melanocyte-specific promoter (Fuse *et al*, 1996), while the other Mitf isoforms are more broadly expressed. To probe for Mitf-M expression in CCS, we initially performed reverse transcription (RT)–PCR analysis of RNA from two CCS cell lines (DTC1 and Su-ccs-1) using Mitf-M-specific primers (Figure 2B). Microphthalmia-associated transcription factor-M RNA is readily detectable in DTC1 and Su-ccs-1 cells, with HeLa cell RNA serving as a negative control (Figure 2B, top panel). We extended the analysis to several additional CCS cell lines and the pigmented melanoma cell line Mel28 (Carey *et al*, 1976) (as positive control) or HeLa cells (as negative control) using an RNAse protection assay (Figure 2B, bottom panel). Using a ^32^P-labelled antisense probe spanning positions +136 to −50 of the Mitf-M promoter (Figure 2A), protected fragments of ∼136 nt representing correctly initiated Mitf-M transcripts are observed in Mel28 cells but not in HeLa cells (as expected) and are observed in all CCS cell lines tested at levels similar to Mel28. Considering the structure of the Mitf gene (Figure 2A), the above result indicates that the Mitf-M promoter is active in CCS cells.

### Expression of Mitf-M protein in CCS cells

Following detection of Mitf-M RNA, we performed Western blotting to examine expression of Mitf proteins in CCS cells compared with Mel28 cells (Figure 3). We used a monoclonal antibody (C5) that detects all Mitf isoforms (Figure 3A) and that has been extensively used for studies of Mitf-M (Bertolotto *et al*, 1998; King *et al*, 1999; King *et al*, 2001; Wu *et al*, 2000). In Mel28 cells, the C5 antibody detects four polypeptides within the reported size range of Mitf (55–75 kDa, Figure 3B) and these polypeptides are absent or expressed at very low levels in nonmelanoma cells (HeLa and Jeg3). The above pattern is consistent with (but more complex than) other studies using melanoma cells and the C5 antibody (Bertolotto *et al*, 1998; King *et al*, 1999; Wu *et al*, 2000) or another antibody (D5) (Hemesath *et al*, 1998) in which an ∼55–60 kDa Mitf-M doublet is detected. Several considerations indicate that in Mel28 cells, either the fastest migrating polypeptide (indicated by an open box in Figure 3B) or the slower migrating doublet (open circles, Figure 3B) or both of the above, are Mιtf-M. First, Mitf-M is significantly smaller than all other Mitf isoforms (Figure 3A) and migrates faster in SDS gels at around 55–60 kDa (Fuse *et al*, 1999). Thus, if the polypeptides detected in Mel28 cells are different Mitf- isoforms, the fastest migrating polypeptide(s) in the range of 55–60 kDa are Mitf-M. Second, Mitf-C (Fuse *et al*, 1999), Mitf-D (Takeda *et al*, 2002) and Mitf-H (Amae *et al*, 1998) are reportedly not expressed in melanoma cells. The slowest migrating polypeptide in Mel28 cells (open triangle, Figure 3B) is consistent with the size of the more widely expressed Mitf-A isoform (Fuse *et al*, 1999). Significantly, with respect to the presumptive Mitf-M isoforms in Mel28 cells (open box and/or open circles), the C5 antibody detected a similar but not identical pattern of polypeptides in all CCS cell lines (see discussion). Although we cannot unequivocally assign Mitf-M to a particular band(s), based on the considerations above, we conclude that Mitf-M (or isoforms thereof) is expressed at significant levels in all CCS cell lines tested. Moreover, unless there is translational control of Mitf-M, the above result is expected based on the significant levels Mitf-M RNA transcripts in all of the CCS cells studied (Figure 2B).

Microphthalmia-associated transcription factor-M functions in melanocyte differentiation and survival, and directly activates the promoters of genes, including tyrosinase, that are required for pigment production (Bentley *et al*, 1994; Hemesath *et al*, 1994; Yavuzer *et al*, 1995; Yasumoto *et al*, 1997). To evaluate the downstream effects of Mitf-M expression in CCS cells, we surveyed expression of tyrosinase (Figure 3B, bottom panel) by Western blotting (using tyrosinase antibody C19, Santa Cruz, CA.). Strikingly, tyrosinase was expressed at significant levels in only one cell line (MST-2) and was either very weakly expressed (Su-ccs-1 and Kao (data not shown)) or was undetectable (DTC1, MST-1 and MST-3) in other CCS cell lines. This result is consistent with a striking pigmentation that is visible with the naked eye for MST-2 cells (KAWL, unpublished observations). In summary, our results show that while Mitf-M is consistently and stably expressed in CCS cell lines in culture, pigmentation, as scored by tyrosinase expression, is generally not preserved.

### Activity of the Mitf-M promoter in CCS cells

**Figure 4:**
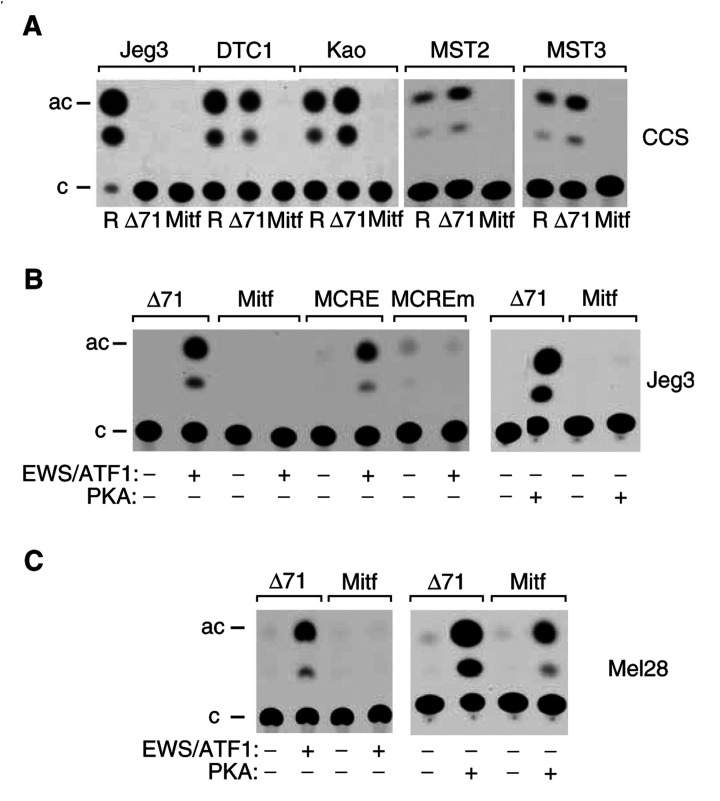
Mitf-M promoter analysis. (**A**) Mitf-M promoter activity in CCS cell lines. The CCS cell lines indicated or Jeg3 cells as control were transfected with pRSVCAT (R), Δ(−71)SomCAT (Δ71) or pMITF-MCAT (Mitf). (**B**) Transactivation by EWS/ATF1 and PKA in Jeg3 cells. Cell were transfected with the reporter plasmids (pΔ(−71)SomCAT (Δ71), pMITF-MCAT (Mitf), pMCRECAT (MCRE) and pMCREmCAT (MCREm)) indicated above in the absence (−) and presence (+) of pΔ287C expressing EWS/ATF1 or pCMVCα expressing the catalytic subunit of PKA. (**C**) Transactivation by EWS/ATF1 and PKA in melanoma cells. Mel 28 cells were transfected with the reporter plasmids (pΔ(−71)SomCAT (Δ71) and pMITF-MCAT (Mitf)) indicated above in the absence (−) and presence (+) of pΔ287C expressing EWS/ATF1 or pCMVCα expressing the catalytic subunit of PKA.

All CCS cell lines used in our study are able to support high levels of constitutive ATF-site- dependent promoter activity in a transient transfection assay (Figure 1B). We used the above assay to ask whether the Mitf-M promoter (pMITF-MCAT) can be activated in CCS cells (Figure 4A

). Surprisingly, however, pMITF-MCAT exhibited near background levels of activity in DTC1, Kao, MST-2 and MST-3 cells, indicating that endogenous EWS/ATF1 is unable to activate a transiently introduced Mitf-M promoter in CCS cells. To test directly the ability of EWS/ATF1 to activate the Mitf-M promoter, we used a previously described transient cotransfection assay in which ATF-dependent promoters (including pΔ(−71)SomCat) are strongly activated by exogenous EWS/ATF1 (Brown *et al*, 1995). Similar to the above result in CCS cells, exogenous EWS/ATF1 (expressed from pΔ287C) is unable to activate the Mitf-M promoter in either Jeg3 cells (Figure 4B) or the melanoma cell line Mel28 (Figure 4C) in which the Mitf-M promoter is normally active. As a positive control for the integrity of the Mitf-M reporter, pMITF-MCAT is able to exhibit a melanocyte-specific cAMP-response in Mel28 cells (Figure 4B and C) as expected (Bertolotto *et al*, 1998). Finally, we tested the ability of the ATF binding site in the Mitf-M promoter to confer EWS/ATF1 responsiveness to a heterologous promoter in Jeg3 cells (Figure 4C). pMCRE contains tandem ATF sites from the Mitf-M promoter placed upstream of the RSV TATA box and is activated by EWS/ATF1, while mutations that eliminate ATF1 binding (pMCREm) also eliminate response to EWS/ATF1. Similar results are obtained in melanoma 501-Mel cells upon fusion of the Mitf-M CRE to the tk promoter (data not shown). Taken together, the above results using transient transfection assays demonstrate that the Mitf-M ATF binding site can confer response to EWS/ATF1 but that surprisingly, the Mitf-M promoter is refractory to EWS/ATF1, even under conditions (in melanocytes) in which the Mitf-M promoter is amenable to activation by cAMP.

## DISCUSSION

The ability of Mitf to induce melanocytic or pigmented features in other cell types (Tachibana *et al*, 1996; Planque *et al*, 1999) suggests that the occurrence of such features in CCS arises due to ectopic expression of Mitf-M. Consistent with this idea, Mitf-M transcripts have been detected in nonviable tumour specimens by RT–PCR (Antonescu *et al*, 2002), indicating that Mitf-M RNA can serve as a diagnostic marker for CCS. Here, we report that the Mitf-M promoter is active and the Mitf-M protein is expressed in several cultured CCS cells. Our results therefore establish a panel of cell lines that provide a valuable experimental resource for studies of CCS and the role of Mitf-M in cellular differentiation and survival. In contrast to Mitf-M, tyrosinase expression is not generally maintained (MST-2 cells are an exception although even in this case, tyrosinase levels are greatly reduced following prolonged culture (KAWL, unpublished observations)). The above results are also similar to those observed in melanoma, whereby amelanotic tumour samples remain positive for Mitf (King *et al*, 1999).The apparent absence of tyrosinase in our experiments is consistent with earlier studies of most CCS cell lines (Epstein *et al*, 1984; Sonobe *et al*, 1993; Liao *et al*, 1996; Hiraga *et al*, 1997), which are only weakly melanotic. In light of our results, the absence of tyrosinase expression in cultured CCS cells is notable, since the tyrosinase promoter is a direct target for Mitf-M in melanoma cells (Bentley *et al*, 1994; Yasumoto *et al*, 1994). The observation that exogenous Mitf-M fails to reactivate the tyrosinase promoter in melanoma cells that have lost expression of Mitf-M (Vachtenheim *et al*, 2001) suggests that Mitf-M might, likewise, not be able to activate the tyrosinase promoter in CCS cells. Alternatively, degradation of tyrosinase protein similar to that which occurs in amelanotic melanoma (Halaban *et al*, 1997) might explain lack of tyrosinase in cultured CCS cells. The above possibilities remain to be examined.

As we have described in the results section, lack of an Mitf-M-specific antibody precludes an unequivocal assignment of Mitf-M among the polypeptides detected in our Western blot analysis (Figure 3B). We note however that dual phosphorylations of Mitf-M by c-kit induced MAP kinase (on S73) and Rsk-1 (on S409) dramatically affect the mobility of Mitf-M on SDS gels and the stability of Mitf-M (Wu *et al*, 2000; Xu *et al*, 2000). Specifically, phosphorylation of either S73 or S409 alone accounts for the 55–60 kDa Mitf-M doublet reported in other studies (Hemesath *et al*, 1998; Wu *et al*, 2000; Xu *et al*, 2000) and an unphosphorylatable S73A/S409A Mitf-M double mutant is greatly stabilised and migrates faster than the 55–60 kDa doublet (Wu *et al*, 2000). Moreover, the relative levels of Mitf-M phosphovariants differ widely in different melanoma cells (King *et al*, 1999), presumably reflecting variation in the MAP/Rsk-1 signalling status. Taking the above observations together, it seems quite possible that in CCS cells, the fastest migrating polypeptide (Figure 3B, open box) and the slower migrating doublet (Figure 3B, open circles) are all phosphovariants of Mitf-M, and that the differential pattern of bands observed in CCS reflects variations in endogenous signalling pathways in the different CCS cells examined.

Our results raise the possibility that Mitf-M is involved in CCS tumorigenesis. The maintenance of Mitf-M expression may reflect a selection process that indicates a role for Mitf-M in CCS cell survival. In this respect, it is pertinent that Mitf-M functions in normal melanocyte survival (Opdecamp *et al*, 1997) by mediating c-Kit signalling (Hemesath *et al*, 1998). The expression of Mitf-M may sensitise CCS cells to a c-Kit/MAP kinase signalling pathway in a manner that contributes to their malignant character. It will be of interest to manipulate Mitf-M expression in CCS cells and examine the effects on cell growth/survival and tumorigenicity in nude mice. If Mitf-M is required for maintenance of CCS tumorigenicity, restricted expression of Mitf-M indicates that it represents a potential therapeutic target for CCS.

While expression of Mitf-M in CCS suggests that EWS/ATF1 may activate the Mitf-M promoter, we have been unable to demonstrate this directly. Our results are equally consistent with either of two possibilities. Firstly, despite the fact that EWS/ATF1 is able to bind to the Mitf-M CRE and activate transcription when out of context (Figure 4), it may simply be the case that EWS/ATF1 is not directly responsible for activation of the endogenous Mitf-M promoter in CCS cells. The inability of EWS/ATF1 to activate the Mitf-M promoter in melanocytes, under conditions in which the promoter is amenable to activation by cAMP (Figure 4), supports this idea. Another alternative is that lack of EWS/ATF1 responsiveness in transient assays might reflect a requirement for an appropriate chromatin context of the Mitf-M promoter. The ability of the Mitf-M ATF binding site *per se* to confer response to EWS/ATF1 in a heterologous promoter context suggests that other transcription factors, including Pax 3, Sox10 and Lef1 that are known to directly regulate the Mitf-M promoter (reviewed in Goding, 2000), might cooperate with EWS/ATF1 to activate the chromosomal Mitf-M promoter in CCS cells.
